# Advances in the application of proteomics in lung cancer

**DOI:** 10.3389/fonc.2022.993781

**Published:** 2022-09-27

**Authors:** Bai Ling, Zhengyu Zhang, Ze Xiang, Yiqi Cai, Xinyue Zhang, Jian Wu

**Affiliations:** ^1^ Department of Pharmacy, The Yancheng Clinical College of Xuzhou Medical University, The First people’s Hospital of Yancheng, Yancheng, China; ^2^ Nanjing Medical University School of Medicine, Nanjing, China; ^3^ Zhejiang University School of Medicine, Hangzhou, China; ^4^ Stomatology Hospital, School of stomatology, Zhejiang University School of Medicine, Zhejiang Provincial Clinical Research Center for Oral Diseases, Key Laboratory of Oral Biomedical Research of Zhejiang Province, Cancer Center of Zhejiang University, Hangzhou, China; ^5^ Department of Clinical Laboratory, The Affiliated Suzhou Hospital of Nanjing Medical University, Suzhou Municipal Hospital, Gusu School, Nanjing Medical University, Suzhou, China

**Keywords:** lung cancer, proteomics, tumorigenesis, diagnosis, treatment

## Abstract

Although the incidence and mortality of lung cancer have decreased significantly in the past decade, it is still one of the leading causes of death, which greatly impairs people’s life and health. Proteomics is an emerging technology that involves the application of techniques for identifying and quantifying the overall proteins in cells, tissues and organisms, and can be combined with genomics, transcriptomics to form a multi-omics research model. By comparing the content of proteins between normal and tumor tissues, proteomics can be applied to different clinical aspects like diagnosis, treatment, and prognosis, especially the exploration of disease biomarkers and therapeutic targets. The applications of proteomics have promoted the research on lung cancer. To figure out potential applications of proteomics associated with lung cancer, we summarized the role of proteomics in studies about tumorigenesis, diagnosis, prognosis, treatment and resistance of lung cancer in this review, which will provide guidance for more rational application of proteomics and potential therapeutic strategies of lung cancer.

## Introduction

Although the incidence and mortality of lung cancer have decreased significantly in the past decade, it is still one of the leading causes of death, which greatly impairs people’s life and health (1). Every year, there is about 2.2 million new cases and 1.79 million deaths according to conservative estimation (2, 3). Lung cancer can be divided into two main types, non-small cell lung cancer (NSCLC) and small cell lung cancer (SCLC), among which NSCLC is the major one (4). As one of the most frequent diagnosed cancers with heterogeneity, there has been always great burden of lung cancer diagnosis. Diagnosis of NSCLC often depends on clinical symptoms like cough, hemoptysis and chest pain, paraneoplastic syndromes, laboratory abnormalities and histologic confirmation, with staging by invasive methods including mediastinoscopy and mediastinal lymph node biopsy, or imaging technology (5). For SCLC, pathological diagnosis with immunohistochemistry and biopsy, and staging with Computed Tomography (CT), Magnetic Resonance Imaging (MRI), and positron emission tomography (PET) are common screening approaches (6). But these methods are sometimes not that accurate, and have limitation in early diagnosis (5).

With the development of proteomics, hundreds of biomarkers have been found. As protein markers can reflect body status better than other kinds of biomarkers such as DNA (7), the explored biomarkers have greatly enhanced the accuracy of diagnosis in several certain diseases (8–10), and reduce some unnecessary invasive biopsies (11). Samples for clinical proteomics can be obtained from biopsy tissues and body fluid especially plasma, saliva, lavage fluid and effusion (5). Besides, proteomics can be integrated with other approaches for better patient stratifications and individual management based on more rational classification and earlier detection (12). However, increasing sensitivity and specificity still need to be improved (13, 14). The treatment of lung cancer consists of traditional radiotherapy, chemotherapy and surgery. Meanwhile, novel therapies including immunotherapy have also been developed. The advances and clinical applications of these new treatments have reinforced the need for accurate sub-classification of lung cancer (15). Furthermore, metastasis, recurrence and resistance are challenges for us.

Proteomics is an emerging technology that involves the application of techniques for identifying and quantifying the overall proteins in cells, tissues and organisms, and can be combined with genomics, transcriptomics to form a multi-omics research model (16). Proteomics focuses on the product of genes, also means the active substance in the cell, so it is dynamic and complex, and is also a good complement to genomics to show functions and interactions of proteins (17). By comparing the content of proteins between normal and tumor tissues, proteomics can be applied to different clinical aspects like diagnosis, treatment, and prognosis, especially the exploration of disease biomarkers and therapeutic targets (18). And the applications of proteomics have promoted the research on lung cancer.

**Figure 1:**
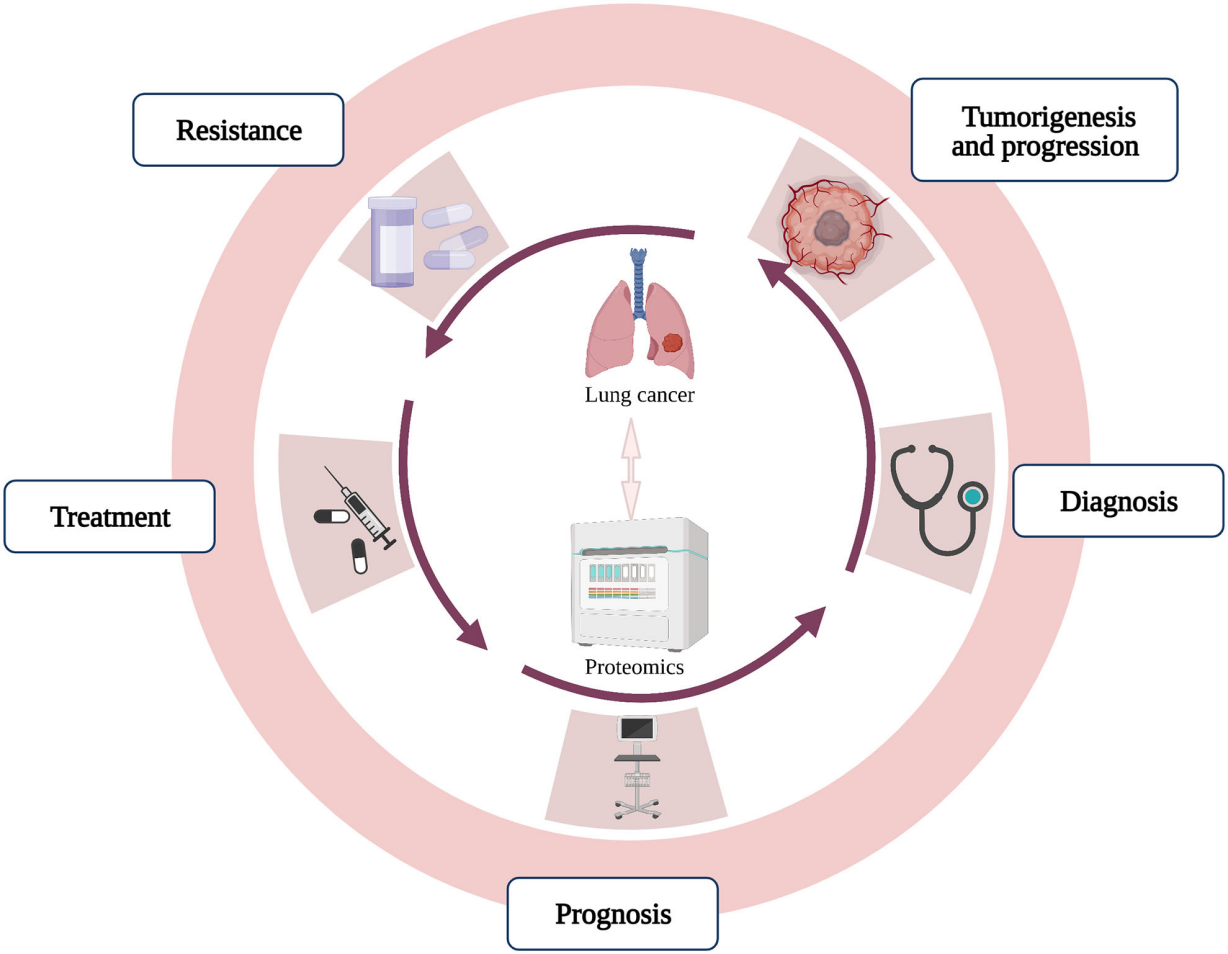
The application of proteomics in lung cancer.

To figure out potential applications of proteomics associated with lung cancer, this review summarizes the role of proteomics in studies about tumorigenesis, diagnosis, prognosis, treatment and resistance of lung cancer, which will provide guidance for more rational application of proteomics and potential therapeutic strategies of lung cancer (**Figure 1**).

## Proteomic technologies

Traditional proteomics employs methods such as protein microarrays and electrophoresis, and the development of mass spectrometry expands the range of application of proteomics.

Electrophoresis includes SDS-PAGE (SDS, sodium dodecyl sulfate; PAGE, polyacrylamide gel electrophoresis), two-dimensional gel electrophoresis (2DE) and two-dimensional fluorescence difference gel electrophoresis (2D-DIGE), which have been widely used despite some obvious disadvantages like low throughput and obscuration of low abundance proteins (19).

Mass spectrometry has promoted the development of proteomics, especially quantitative proteomics. Technically, mass spectrometers are mainly composed of the ionization source, the mass analyzer, and the detector. In 1998, Tanaka, K. et al. first analyzed large molecules like proteins using matrix-assisted laser desorption ionization (MALDI) and a time-of-flight (TOF) mass spectrometer, which was named MALDI-TOF-MS technology later (20). While surface-enhanced laser desorption ionization with time-of-flight mass spectrometry (SELDI-TOF-MS) has become popular in the examination of protein expression differences in clinical settings because of its ease of use and good throughput ability (21). MALDI and SELDI are two most common platforms for mass spectrometry technology. However, because of the limitation of 2DE, MALDI and SELDI in proteome coverage, sensitivity and resolution, they have been gradually superseded by liquid-chromatography/mass spectrometry (LC/MS), which can assist other strategies for quantification (22).

Most of the time, quantitative proteomics can be divided into two main categories, labeled and label-free approaches. Labeled approaches include *in vivo* labeling and *in vitro* labeling. Stable isotope labeling by amino acids in cell culture (SILAC) is a simple, inexpensive, and accurate *in vivo* labeling approach, which can be applied in any cell culture system (23). For *in vitro* labeling, isobaric tag for relative and absolute quantitation (iTRAQ) shows greater capability to identify high abundance proteins (24), and tandem mass tags (TMT) allow identification of peptides from different samples with better ease and accuracy (25). Data acquiring methods used for quantification are mainly data-independent acquisition (DIA) and data-dependent acquisition (DDA). DIA usually employs spectral library searches, while DDA workflows are mostly based on sequence database searches (26). To summarize, more and more mature proteomic technologies allow better application of proteomics in researches related to lung cancer.

## Role in the tumorigenesis and progression of lung cancer

The application of proteomics methods has substantially improved our knowledge about the genesis and progression of lung cancer. Some pathways or essential molecules associated with lung cancer were gradually identified through proteomics (**Table 1**). A study operated the foundational and differential proteomics revealed that βIII-tubulin, an isotype of β-tubulin expressed in neural tissues, which was believed to correlate with chemoresistance and poor survival, might be regulated by the PTEN/AKT axis to influence tumor proliferation and metastasis, and mspin, a tumor suppressor related to adherence was observed to increase significantly in TUBB3 knock-down NSCLC cells, accompanied by changes in cell morphology (27). Chen et al. found that USP9X could facilitate the genesis of NSCLC through the dual specificity protein kinase TTK, also known as MPS1 (28), a key regulator of the pindle assembly checkpoint which maintains genomic integrity (33). Besides, after analyzing 108 primary tumor tissues and 99 paired normal lung tissues from patients, Satpathy et al. described a proteogenomic landscape of LSCC, and identified NSD3, part of NSD family which plays a key role in chromatin regulation, as an alternative driver in LSCC with FGFR1 amplification (34). In addition, the early molecular events of lung cancer also deserve attention. In a study, proteomics was used to examine tracheal epithelial cells in bronchial brushing specimens of 15 people at different risk of lung cancer obtained from main stem bronchus when performing bronchoscopy. The total of 237 up-regulated proteins and 75 down-regulated proteins were identified, and subsequent Kyoto Encyclopedia of Genes and Genomes (KEGG) analysis proved that the key enzymes in the glycolysis pathway, the tricarboxylic acid cycle, the pentose phosphate pathway, and the galactose and glycogen metabolism were all overexpressed, indicating that early events of metabolic reprogramming may occur in the cytologically normal bronchial epithelium of individuals at high risk of lung cancer. From the low-risk to high-risk group, the expression of proteins has a notable increase trend, including GLB1, PYGB, PGM1 and so on. These very early molecular events in lung cancer can help us to understand the occurrence of lung cancer further and provide new ideas for developing chemoprevention strategies (29).

**Table 1:** Role in the tumorigenesis and progression of lung cancer.

Authors	Year	Type of lung cancer	Related molecules	Related pathways	Functions
McCarroll et al. (27)	2015	NSCLC	βIII-tubulin	PTEN/AKT signaling pathway	Promotion of metastasis.
Chen et al. (28)	2018	NSCLC	TTK	Proteasomal degradation pathway	USP9X substrate to promote tumorigenesis.
Rahman et al. (29)	2016	–	GLB1, PYGB, PFKL, PGK1, LDHB, IDH1, IDH2, and ME2	Carbohydrate metabolic pathway	Very early molecular events in lung tumorigenesis and metabolic reprogramming.
Chang et al. (30)	2012	LUAD	Alpha-1 Antitrypsin	–	Metastasis-promoting secretory protein.
Min et al. (31)	2014	NSCLC	ITGA2, MAPK1, ACTN4, FLNA, and FLNB	Aminoacyl-tRNA biosynthesis pathway; Pentose phosphate pathway; Proteasome pathway; Arginine and proline metabolism pathway; DNA replication pathway; Focal adhesion pathway.	Promotion of metastasis.
Pal et al. (32)	2021	NSCLC	FLNC, DSE, CPA4, TUBB6, and BICC1	ECM organization pathway; Cell adhesion pathway; Cell migration pathway.	Migration control factors.

NSCLC, non-small cell lung cancer; LUAD, lung adenocarcinoma.

The metastasis of lung cancer is another important clinical issue. To explore the relevant mechanism of lung cancer metastasis, Chang et al. compared two groups of lung adenocarcinoma (LUAD) cell lines, low-metastatic CL1-0 and high-metastatic CL1-5 through proteomics, and they identified 68 proteins with different expressions in these two groups. The overexpression of A1AT in CL1-0 might significantly increase its invasiveness, while the expression of A1AT in CL1-5 was down-regulated, thereby reducing its invasiveness. This suggested that A1AT, a hydrolytic enzyme, may be closely related to the invasion and metastasis of LUAD cells (30). After a proteomics study by label-free quantitative analysis and N-terminal analysis on two groups of human NSCLC cell lines with different metastasis potentials, NCI-H1703 (primary cell, stage I) and NCI-H1755 (metastatic cell, stage IV), 11 quantitatively expressed proteins and 8 N-terminal peptides were identified with differential expression, and were enriched mostly in the adhesion-related pathways, which can determine the possible biomarkers for NSCLC metastasis (31). Besides, some systems were built to evaluate metastasis potentials, such as the MigExpress system, which was proposed to be used to assess NSCLC-associated migration factors and provide more comprehensive transcriptome and proteomic data related to NSCLC metastasis (32).

## Role in the diagnosis of lung cancer

Proteomics can be used to discover new lung cancer biomarkers that can be applied to the diagnosis (**Table 2**). The related techniques have also been developed. Sun et al. used serum functional enzymes enriched in the glycolytic pathway in lung cancer patients to verify the Protein Elution Plate PEP technology combined with mass spectrometry, which can systematically monitor serum functional enzymes and may become a substitute method for biomarker sequence annotation (35).

**Table 2:** Role in the diagnosis of lung cancer.

Authors	Year	Type of lung cancer	Related molecules	Related pathways	Functions
Sun et al. (35)	2016	–	Glyceraldehyde-3-phosphate Dehydrogenase, Pyruvate Kinase and Enolase 1	Glycolytic pathway.	Alternative to sequence annotation for diagnosis.
Sun et al. (36)	2017	–	Annexin, Zinc-alpha-2-glycoprotein, MUC5B, Histone H3, CD5 antigen-like, Alpha-1-acid glycoprotein 1, Integrin beta-2, Carbonic anhydrase 6, heat shock 70 kDa protein 4, and deleted in malignant brain tumors 1 protein	Integrin signaling pathway; Inflammation mediated by chemokine and cytokine signaling pathway; Cytoskeletal regulation by Rho GTPase pathway.	Diagnostic marker from circulating exosomes proteome.
Wang et al. (37)	2018	NSCLC	lipopolysaccharide-binding proteins	Metastasis-related pathways.	Serum exosomal biomarkers to predict metastasis.
Boccellino et al. (38)	2019	LUAD	AMBP, α2 macroglobulin, and A1AT	–	Biomarkers for early detection.
He et al. (39)	2007	NSCLC	α-enolase, carcinoembryonic antigen, and cytokeratin 19 fragment	Glycolysis pathway.	Combination for sensitive diagnosis.
Patz et al. (40)	2007	–	Carcinoembryonic antigen, retinol binding protein, 1-antitrypsin, and SCC antigen	–	Diagnostic panel.
Li et al. (41)	2012	LSCC	Cathepsin D preproprotein, mitochondrial heat shock 60 kDa protein 1 variant 1	Proteolytic degradation pathway; Cell invasion pathway; Apoptosis pathway.	Potential diagnostic markers.
**Authors**	**Year**	**Type of lung cancer**	**Related molecules**	**Related pathways**	**Functions**
Kim et al. (42)	2007	LSCC	SCCA1, SCCA2, S100A8, S100A9, annexin I, and annexin II	–	Potential markers of early squamous metaplastic or precancerous changes.
Ahn et al. (43)	2014	SCLC	APCS, C9, SERPINA4, and PON1	–	Diagnostic markers.
Zhou et al. (44)	2013	LUAD	Tyrosyl-tRNA synthetase, Microtubule-actin cross-linking factor 1	–	Diagnostic markers and the hematogenous metastasis predictors.
Sung et al. (7)	2018	–	Quiescin sulfhydryl oxidase	–	Metastatic markers
Guergova-Kuras et al. (46)	2011	NSCLC	LRG1, ACT, Hpt, C9, and CFH	–	Diagnostic panel.
Hsu et al. (47)	2016	LUAD	ERO1L, PABPC4, RCC1, RPS25, NARS, and TARS	Translation_Regulation of translation initiation pathway; Aminoacyl-tRNA biosynthesis in cytoplasm pathway; Tricarbonic acid cycle pathway.	Diagnostic markers

NSCLC, non-small cell lung cancer; LUAD, lung adenocarcinoma; LSCC, lung squamous cell carcinoma; SCLC, small cell lung cancer.

In recent years, exosomes have received more and more attention in the exploration of tumor biomarkers (47). Sun et al. used label-free quantification to compare protein profiling in serum and saliva exosomes in healthy people and patients with lung cancer, and they found that 11 proteins with significant differences in both body fluids seem to serve as biomarkers for lung cancer diagnosis. These results also supported the hypothesis that circulating exosomes contain disease-associated proteins and can be detected in body fluids (36). Comparing the metastatic and non-metastatic NSCLC exosomes, Wang et al. found significant differences in the expression of lipopolysaccharide-binding proteins, and these proteins may be a driver of NSCLC metastasis, which can be used as potential biomarkers of NSCLC metastasis and therapeutic targets (37).

Some identified proteins can be used for better diagnosis and differentiation of lung cancer. AMBP, α2 macroglobulin and A1AT were shown to be endopeptidase inhibitor, which can promote the development of lung cancer. Boccellino et al. analyzed these three proteins quantitatively in the serum samples from 20 NSCLC patients and 10 health controls through multiple reaction monitor. AMBP can be lysed *in vivo* into two chains: α-1-microglobulin and inter-α-trypsin inhibitor light chain (also named bikunin). In advanced cancer, bikunin levels were elevated, A1AT levels were reduced, and the levels of α2 macroglobulin appeared to be independent of cancer stage, suggesting that these three proteins may be used to grade NSCLC in an early stage (38). Based on the proteomics methods, autoantibodies against α-enolase were considered potential biomarkers of NSCLC, and the combined detection of autoantibodies against α-enolase, carcinoembryonic antigen and cytokeratin 19 fragment could significantly improve the sensitivity of NSCLC diagnosis (39). Bouamrani et al. used surface-enhanced laser desorption ionization-time-of-flight mass spectrometry technology to perform *in situ* proteomic profiling in mouse and human lung cancer tissue samples, and they proved that this kind of direct tissue proteomic could distinguish glioblastomas from oligodendrogliomas effectively, suggesting the possible application of direct tissue proteomic analysis in lung cancer diagnosis (48). Patz et al. attempted to use the expression data of proteins in serum samples of 100 lung cancer patients as a training set, and they finally selected a group of proteins, including CEA, RBP, SCC, and A1AT. The combination of these four proteins showed desirable accuracy for the detection and diagnosis of lung cancer (40). The Tokyo Medical University Hospital in Japan and the Lund University hospital in Sweden also proposed to use the proteomics sequencing platform to distinguish neuro-endocrine lung cancer phenotype patients from SCLC and large cell lung cancer patients better, which also provided the targeted early treatment strategies (49).

In recent days, screening and early detection of lung cancer has gained great attention to improve the survival and prognosis of patients, and proteomics made some contributions and provided potential early-stage diagnostic biomarkers. Exhaled breath condensate of 192 individuals including 48 different types of lung cancer patients was analyzed by proteomics, and indicated that cytokeratins might be potential biomarkers for early detection of lung cancer (50). Besides, Jiang et al. developed an effective platform based on the proteomic analysis of salivary samples collected from 89 early lung cancer patients, 11 advanced lung cancer patients and 50 healthy volunteers. The sensitivity and specificity were high enough to distinguish early cacer patients from healthy people (51). Pan et al. also constructed a panel of p53, HRas, and ETHE1 using a 2-phase strategy for early detection of lung cancer (52). Currently, NSCLC still lacks early detection markers, and always advances when diagnosed, which makes NSCLC a malignant disease with poor survival and prognosis. However, proteomics might help change this situation. Using agrocybe aegerita lectin 2 that has high affinity to GlcNAc (AALNL/AAL2) to enrich serum glycopeptides, PON1 was identified to differ between Stage I NSCLC and healthy controls in serum samples from 120 enrolled participants including 58 healthy, 25 benign, and 37 NSCLC subjects (53). Among subtypes of NSCLC, early diagnosis of LSCC has been focused on in recent days. The cell membrane proteins taken from tumor tissues and normal bronchial epithelial tissues in 10 patients with non-metastatic LSCC were examined by matrix-assisted laser desorption ionization-time of flight mass spectrometry, and 12 proteins were up-regulated while 7 downregulated in tumor tissues. Among the total of 19 candidate markers, CTSD and HSP60 were further verified by the western blotting, which may serve as new markers for early diagnosis of LSCC (41). Additionally, Kim et al. used chromatography-tandem mass spectrometry to examine apical surface fluid of squamous metaplastic normal human tracheobronchial epithelial (NHTBE) and mucous NHTBE cells, and eventually they verified 6 differentially expressed proteins, especially SCCA1 (a cysteine proteases inhibitor) and SCCA2 (both serine and cysteine proteases inhibitor), which were expressed only in squamous metaplastic NHTBE cells. These candidate proteins might be used for early diagnosis of LSCC (42). Furthermore, four fucosylated proteins, including APCS, C9, SERPINA4, and PON1, were identified through multiple reaction monitoring-mass spectrometry by Ahn et al., which were proved to be of high value for SCLC diagnosis. In the plasma of SCLC patients, the level of PON1 was significantly reduced, while the fucosylation level of PON1 was significantly increased, suggesting that PON1 and its fucosylation level can be used as a diagnostic marker of SCLC (43). There also exist researches studying early diagnostic protein markers for LUAD, by analyzing lung tissue samples from different stages, a group of six candidate proteins including ERO1L, PABPC4, RCC1, RPS25, NARS, and TARS shown potential for early detection of LUAD (46).

For LUAD, more detection markers have been found, and Zhou et al. identified differentially expressed proteins in LUAD tissues and corresponding normal bronchial epithelial tissues from 7 patients. The mass spectrometric analysis found that 13 proteins were up-regulated and 9 proteins were down-regulated, of which two possible biomarkers, TyrRS and MACF-1, were identified by immunohistochemistry (44). The specific clinical application needs to be further explored. Meanwhile, QSOX1 was another biomarker validated by mass spectrometry to be significantly up-regulated in tumor tissues than in nearby normal tissues in lung cancer patients, and further studies found that QSOX1 can promote the metastasis potentials of lung cancer cells (7). Guergova-Kuras et al. used monoclonal antibody proteomic to analyze the plasma proteomes of 4 NSCLC clinical cohorts, and 13 lung cancer-related monoclonal antibodies were identified, and a total of five homologous proteins were selected. In two independent clinical data sets, a panel of these 5 biomarkers showed high sensitivity and specificity in diagnosing stage I NSCLC, and the combination of the panel of 5 biomarkers and a known cancer marker CYFRA could better improve the efficacy in diagnosis (45).

Besides, there exist specific differences based on sex in both clinical and molecular patterns, although these are always neglected. In NSCLC, better survival has been observed in women (54, 55), which might be related to the sex-biased differences especially those in expression of some functional proteins. Sex-based biomarkers can enhance the accuracy of diagnosis, Izbicka et al. explored plasma biomarkers in NSCLC patients with the help of multiplex immunoassays and mass spectrometry, and found that in male, sFas, MMP-9, and PAI-1 had higher expression, while in female, sCD40 had higher expression compared to that of healthy volunteers (56). Attention has also been focused on different immune state between male and female (57). It has been known that women usually have a stronger immune response than men, which can influence the tumorigenesis, diagnosis, treatment and prognosis. Ramsey et al. identified the level of 171 serum proteins in 1,676 participants and variations exist in the concentration of 56% of biomarkers between female and male, which was believed to contribute to deeper research (58). As relevant information are still limited, more researches are needed to explore sex-specific biomarkers with proteomics technology.

## Role in the prognosis of lung cancer

Proteomics can have an important role in predicting the prognosis of patients with lung cancer, primarily by identifying expression of certain proteins. Dingemans et al. applied MALDI to successfully predict the survival outcomes based on pre-treatment serum samples in patients with phase IIIb or IV NSCLC accompanied with KRAS mutation (59). Additionally, HSP 90β was identified as a potential prognostic blood biomarker for LUAD based on mass spectrometry and was validated in an independent cohort of 705 LUAD patients and 282 healthy controls (60).

Proteomics can also be used to predict the possible metastasis of lung cancer. Liu et al. applied proteomics to non-metastatic 393P and metastatic 344SQ NSCLC cell lines to analyze protein expression differences in extracellular vesicles (EVs), and they demonstrated that Tspan8 was selectively highly expressed in 344SQ NSCLC cell lines. It was also confirmed that the abnormally high expression of Tspan8 can promote the metastasis potentials of NSCLC cells. Therefore, Tspan8 may be an ideal marker for predicting distal metastasis in patients with NSCLC (61). Moreover, the mitochondrial proteomic analysis of the high-metastatic large cell lung cancer cell line L-9981 and the low-metastatic NL-9980 was conducted. A total of 217 differentially expressed proteins were detected, of which 64 proteins with the most significant changes were further enriched and analyzed, indicating that these proteins were mostly related to redox reaction. This also suggested that metastasis of large cell lung cancer can be predicted by establishing a specific dataset of mitochondrial proteins (62).

## Role in the treatment of lung cancer

Proteomics plays a very important role in the treatment of lung cancer. Based on the existing drugs, proteomics can help develop new targets, thereby promoting the advancement of lung cancer treatment strategies.

When it comes to the treatment of SCLC, proteomics is often used for identification and selection of possible targets. As a clinical common-used drug, bortezomib (BTZ) was found to induce the expression of an anti-apoptotic protein MCL-1 in 6 common human SCLC cell lines using a comprehensive proteomics analysis, thereby limiting its clinical efficacy. Otoclax (OBX) could cause obvious growth inhibition and apoptosis of SCLC when it was used with the combination of BTZ (63). The efficacy of this combination therapy needs more clinical trials for further assessment. Also, proteomics help identify potential targets for some clinical drugs. As a PARP inhibitor, the potent anti-SCLC drug talapoarib has its classical target PARP1, but through the chemical proteomics, it was found that unlike other PARP inhibitors, talazoparib might has another special target PARP16. The silencing of PARP16 could significantly inhibit cell survival, especially with the inhibition of PARP1. This enriched our knowledge about the possible mechanism behind the activity of talasoparib and provided a potential new therapeutic target PARP16 (64). A great number of possible therapeutic targets are gradually identified by utilizing proteomics. Coles et al. identified a total of 20 candidate kinases that were more active in SCLC cells than in normal lung or NSCLC cells. Among them, PKA was active to promote propagation of tumor cells in most SCLC cases, and the broad proteomic analysis figured out corresponding signaling networks in SCLC (65).

The management and monitoring of patients can be improved with certain proteomics methods. The preliminary studies showed that matrix-assisted laser desorption/ionization proteomics analysis could stratify SCLC patients and distinguish patients with disease control from those with progressive disease. Therefore, patients who could most benefit from EGFR tyrosine kinase inhibitor (TKI) will be identified (66). Dose-related efficacy of clinical drugs can be evaluated when methylation and acetylation levels of histones in cancer cells after chemotherapy were quantitatively determined using proteomics. For NSCLC, proteomics greatly advanced the development of its treatments. In terms of NSCLC radiotherapy, a study showed that two proteins, CRP and LRG1, which shown great changes in expression during radiotherapy, can be used to stratify patients early in radiotherapy, thereby providing more specific treatment for patients at different stages (67).

The application of proteomics can further explore the specific mechanisms of some drugs. Tivantiniba inhibits the metastasis potentials and deterioration of cancer cells by inhibiting the receptor tyrosine kinase c-MET, but unlike other inhibitors, it inhibits the activity of most NSCLC cell lines, indicating that its activity in NSCLC is not produced only by inhibiting c-MET. Remsing et al. applied an unbiased, mass-spectrometry-based, chemical proteomics approach, and they discovered two new targets of tivantiniba, GSK3 α and β, whose influences in NSCLC didn’t seem to overlap. Inhibiting them at the same time induced apoptosis more effectively in consequent experiments (68). AZD1775 has also shown monotherapy activity against NSCLC, although the underlying mechanism is unclear. An unbiased mass spectrometry-based chemical proteomics approach validated a new target, polo-like kinase 1, which might contribute to the anti-cancer activity of AZD1775 as a monotherapy when targeted with WEE1 simultaneously. Attention should be paid to its limitations as a highly selective WEE1 probe molecule when developing clinical anticancer application of AZD1775 (69).

Periplocin inhibits the growth of lung cancer cells both *in vivo* and *in vitro*, to explain its possible anti-cancer mechanism. Lu et al. found a total of 29 down-regulating proteins and 10 up-regulating proteins in periplocin-treated A549 cells compared to control groups, and the most important related function of these 39 proteins were transcription and proteolysis, which provided new insights for the use of periplocin on the treatment of lung cancer (70). Upon the knock-down of KMT9α (component of histone lysine methyl transferase KMT9) in A549 cells, RNA sequencing and mass spectrometry analysis revealed 460 target genes that were differentially expressed in both mRNA and protein levels, which were mainly enriched in the cell death, regulation of proliferation, as well as regulation of cell cycle pathways, and thus inhibited the proliferation of lung cancer cells and induced non-apoptotic cell death (71). Inhibitors targeting KMT9 might become a new kind of clinical agents. Abraxane (Abr) is an albumin-bound nanoparticle drug that was found to be more effective than pacificaxel (PTX) when used to treat NSCLC. In an experiment related to proteomics, the expression of only one protein, GNA1 was significantly changed in Abr and PTX-treated A549 cells. The decrease of this protein may inhibit growth and impair adhesion function of cancer cells (72). Through the mass spectrometry, Colzani et al. identified six new possible candidate kinase targets for anti-cancer multi-kinase inhibitor E-3810 on human NSCLC cells. Among them, the phosphorylation and activity of DDR2 was experimentally proved to be inhibited by micromolal dose of E-3810 in lung cancer cells overexpressing DDR2. Moreover, E-3810 in HCC-366 cells containing DDR2 mutations could also inhibit the proliferation of cancer cells. This helps us gain a deeper understanding of the mechanism behind the activity of E-3810 (73). Some molecules that are difficult to be exploited as direct drug targets may be proved to have significant influences on druggable molecules using proteomics. Nuclear receptor NR0B1 was identified as a potential druggable molecule for lung cancer dependent on the transcription factor NRF2 (74).

Further exploration of drug efficacy can be another application of proteomics. Huang et al. found that benzethonium chloride (BZN), an anti-infective drug might have some anti-cancer activities clinically because it led to the increase of 60 proteins and the decrease of 179 proteins in A549 cells, many of which are involved in cell cycle regulation (75). In a set of proteomics data, it was observed that RhoGDIα was significantly down regulated in A549 cells treated with 7,8-diacetoxy-4-methylcoumarin (DAMTC), and RhoGDIα regulated RhoA, Rac1 and Cdc42, thus affecting the formation of the cytoskeleton, which is closely related to the morphology, movement, adhesion and other functions of the cells. Therefore, DAMTC may block the migration and angiogenesis of cancer cells, with potential anti-cancer effects (76). OSU03013 was first found in proteomic studies to cause dephosphorylation of GSK3β in A549, CL1-1, H1435 cells, demonstrating the possibility of OSU03013 treating lung cancer (77). Proteomics also provides new ideas in the treatment of metastatic lung cancer. Integrated metabolomics and proteomics demonstrated that the activation of the Wnt/NR2F2/GPX4 axis in lung cancer patients with brain metastasis. Compared with primary lung cancer, the consumption of glutathione was high and two proteins (GPX4 and GSTM1) were up-regulated, which inhibited ferroptosis, and induced chemotherapy resistance and led to poor prognosis consequently. Experiments indicated GPX4 inhibitors could greatly improve the anti-cancer effect of platinum drugs on lung cancer patients with brain metastasis, which needs to be further verified (78). In cancer metastasis and development, cell-generated small extracellular vesicles (sEVs) are of great significance, there seem to have great relations, but with unclear mechanisms. The quantitative proteomics was performed on sEVs produced by highly metastatic lung cancer cells, and sEVs-HGF was recognized as a possible metastatic-related protein. Further studies showed that there existed a synergistic effect between sEVs-HGF and its transmembrane receptor c-Met, suggesting that HGF/c-Met pathway may be a potential therapeutic target for inhibiting lung cancer metastasis (79).

The applications of proteomics can provide more possibilities for drug combination. The phenotypic drug screening proved that midostaurin has strong activity independent of PKC in NSCLC cells. Ctortecka et al. discovered numerous targets and corresponding pathways for midostaurin, such as TBK1, PDPK1, and AURKA, as well as PLK1. In combination with PLK1 inhibitors like BI2536, midostaurin seemed to produce a strong synergistic effect. The study provided a comprehensive insight into midostaurin and enhance the rational design of the combination approach to midostaurin (80). Dasatinib is another clinical drug for NSCLC. To explore the mechanism underlying the anti-cancer activity of dasatinib, chemical proteomics and immunoaffinity purification were combined, and 40 possible kinase targets were finally identified, including SFK members, non-receptor tyrosine kinases, and receptor tyrosine kinases, which could contain functional targets of dasatinib and provide possible combination therapy options (81).

The efficacy of treatment needs to be screened and predicted for better treatment and management of patients. For chemotherapy, in a study, serum MALDI was used to successfully predicted prognosis of NSCLC patients after the treatment of erlotinib (82). Patients accepting radiotherapy can sometimes develop lung toxicity, with multiplex quantitative proteomics approaches to examine platelet-poor plasma obtained from 57 eligible NSCLC patients after radiotherapy, C4BPA and VTN were significantly identified to be up-regulated in patients with grade 2 radiation-induced lung toxicity, while immunoglobulin kappa chain V-III region Ti and region HAH were opposite. The further analysis was conducted to develop three models with better accuracy, including VTN alone, C4BPA + VTN, and C4BPA alone to help predict the occurrence of later grade≥2 radiation-induced lung toxicity (RILT2) (83). In terms of surgical efficacy prediction, proteomics also has certain application value, early recurrence of LSCC after surgery is an important factor for the poor prognosis. With proteomics, Wu et al. figured out that DDX56, one of the members of DEAD-Box Helicase (DDX) family, increased significantly in a group of primary tumor tissues of 20 patients with early recurrent LSCC. Through further pathway enrichment analysis and cytology experimental studies, it was found that DDX56 might influence the incidence of recurrence and survival by miRNA-mediated post-transcriptional regulation of the Wnt signaling pathway (84). A panel of genes was selected by Sharpnack et al., including SUMO1, PCBD1, PSMC5, ARCN1, PPA2, and SRI. They combined corresponding RNAs and proteins to form integral biomarkers, which can better predict the recurrence of LUAD after surgery (85).

Some datasets constructed by proteomics are used for evaluation of treatments. Chen et al. collected tumor tissues and matched normal adjacent tissues (NATs) from 103 untreated lung cancer patients, and built a comprehensive proteomics landscape of east Asian non-smoking LUAD populations to provide strategies and candidate biomarkers for patient stratification, intervention and treatment management (86). Another research attempted to combine different kinds of techniques to complete multiomics analysis and build a dataset on the basis of 110 LUAD tissues and 101 corresponding normal tissues, which may have a promoting effect on the treatment of LUAD (87). Furthermore, an NSCLC subtype classification strategy based on DIA-MS was validated in a cohort of 208 NSCLC patients for better management of late-stage NSCLC patients management (88).

## Role in the mechanisms of resistance

In the clinical treatment of lung cancer, drug resistance is one of the issues that need to be solve urgently, and proteomics plays an important role in exploring the mechanisms of drug resistance. Some pathways have been found to be associated with radiotherapy and chemotherapy resistance to lung cancer. For example, the activation of the PI3K/AKT pathway may be associated with SCLC chemoradiotherapy resistance label-free through mass spectrometry-based proteomics combined with whole-exome sequencing (89).

Cisplatin is a classic drug for lung cancer, and is also one of the drugs prone to drug resistance. Böttger et al. constructed a kind of RPF mouse model by overexpressing the carcinogen Nfifib based on the classical SCLC RP mouse model, and they treated the RPF mice with vector and cisplatin. Comparing lung tissue samples of the vector-treated mice (V-RPF) and cisplatin-treated RPF-Rep mice, a total of 274 differentially expressed proteins were identified, of which 101 were more abundant in RPF-Rep samples, CDH1 in particular, which induced cisplatin resistance by mediating cell-to-cell contact to upregulate the PI3K-AKT pathway. Moreover, 173 differentially expressed proteins were reduced in RPF-Rep samples and a wide set of neuronal differentiation and migration-associated genes were significantly reduced, indicating that cisplatin therapy may induce the transfer to less pronounced neuronal phenotype (90). At the cellular level, 157 upregulated proteins and 140 down-regulating proteins were identified in the cisplatin-resistant A549 cells through quantitative proteomic analysis of cell membrane proteins. Further GO and KEGG analysis showed that proteins up-regulated were mostly associated with enhanced cell adhesion function, thereby enhancing the resistance to cisplatin while those down-regulated tended to be related to signal transduction and cell migration. In addition, LRRC8A, which greatly influences cisplatin uptake, was also downregulated, which reduced cisplatin uptake considerably and was also involved in the generation of cisplatin resistance (91). And in another study, the SILAC proteomics identified high expression of GRP78 and other proteins associated with anti-apoptotic and/or autophagy promotion, which might also contribute to cisplatin resistance (92). Milone et al. performed proteomic approaches to compare the constructed cisplatin-resistant A549 cell line and its parents, and found 13 up-regulated and 2 down-regulated proteins. The interaction networks showed that these proteins may contribute to the development of drug resistance by influencing protein folding and endoplasmic reticulum stress, and consequent increased NSCLC cell aggressiveness (93). Some molecules related to cisplatin resistance mechanism can also be used as a predictor of prognosis of cisplatin treatment. After a 2-DE analysis to compare A549 and cisplatin-resistant A549/DDP cells quantitatively, 9 up-regulated and 3 down-regulated proteins were identified, and DJ-1 was selected for further verification with the western blotting. Then 67 clinical cases were grouped according to the expression of DJ-1. Interestingly, the group with high DJ-1 expression was found to have a significant decrease in overall survival, while the silencing of DJ-1 in A549/DDP cells could partially rescue the responsiveness to cisplatin, and hence DJ-1 might contribute to cisplatin resistance and could also be a potential predictor of prognosis of cisplatin therapy (94).

EGFR inhibitors are often used to treat NSCLC, but some patients inevitably develop resistance to them, and proteomics found that oncogenic transcription factor BCL6 inhibited the transcription of multiple target genes, thereby resulting in reduced apoptosis, which suggested that BCL6 may be an important target for EGFR inhibitor resistance. The consequent experiments demonstrated that targeting both EGFR and BCL6 at the same time would have a potent synergistic effect on killing lung cancer cells, which also provided new combination therapy regimens (95). Osimertinib is a third-generation EGFR TKI for EGFR T790M mutation. However, resistance has still developed. By comparing the proteome and phosphoproteome of mutated LUAD cells sensitive and resistant to osimertinib, the overall proteomic changes of three generations of EGFR TKI resistance were identified, in which mass spectrometry showed that epithelial-mesenchymal transition (EMT) may be associated with osimertinib resistance. In addition, significant changes at important phosphorylation sites on kinases in drug-resistant cells might be part of the mechanisms underlying the generation of the resistance (96). In cell lines resistant to EGFR TKIs, Terp et al. applied spectrometry-based proteomics, and they found that the expression of FGFR1 was significantly increased, and the Akt pathway was abnormally activated. The combination of EGFR and Akt inhibitors might be a possible clinical treatment strategy (97). A group of data from quantitative proteomics, which examined pleural effusions in advanced LUAD patients with EGFR TKI resistance and control group, selected 15 preliminary candidate proteins for further study. The content of CDH3 in the pleural effusion of resistant patients was significantly increased, and influenced the progression-free survival and overall survival. Therefore, CDH3 may be associated with EGFR TKI resistance (98).

Abraxane is nanoparticle form of PTX, after comparing the protein expression profiles of A549 cell lines sensitive and resistant to abraxane by combining quantitative proteomics and GO analysis, these results demonstrated that most of the up-regulated proteins in resistant cells were predominantly enriched in lipid biosynthesis and amino acids metabolism-related pathways, while down-regulated proteins were primarily associated with cytoskeleton and cell adhesion. Some of these proteins were not discovered in previous studies about PTX resistance and this study provided new ideas for following researches on the resistance of nanoparticle form drugs (99). Tufo et al. performed an unbiased approach to detect endoplasmic reticulum proteins in NSCLC A549 cell lines resistant to long-term cis-diaminedichloroplatine(II) (CDDP), and they found significant up-regulation of PDIA4 and PDIA6, which possibly mediated CDDP resistance (100). Microtubule interacting agents (MIAs) are commonly used in the clinic for oncology treatment, but some lung cancer patients have gradually developed resistance to MIAs. Albrethsen et al. applied proteomic analysis to A549 cell lines, a taxol resistant cell line, and an epothilone B resistant cell line. Galectin-1 was found to be up-regulated considerably, indicating that galectin-1 might contribute to the development of resistance to microtubule stabilizer (101).

## Conclusions

Proteomics is a necessary piece of the puzzle of multi-omics, which has played an important role in lung cancer-related study, with great value and application prospects. Proteomics techniques can be combined with a variety of other technologies, when combined with molecular technologies, further exploration of mechanisms will be attained, and protein landscape or related dataset can be built with the combination of computer technologies, thus synthesizing different information to form a comprehensive network. However, it should be noticed that there are still some problems in the application of proteomics. For example, how to further improve the sensitivity and specificity of biomarkers of diagnosis, and how to determine the optimal model in prognosis prediction. The solution to these issues depends not only on the development and innovation of proteomics techniques, but also on combination of technologies and experiments. More studies are needed to further advance the applications of proteomics in lung cancer.

This article reviewed application of proteomics in lung cancer in the last twenty years, and paid attention to relatively comprehensive aspects including tumorigenesis, diagnosis, prognosis, treatment and resistance of lung cancer. And we discussed this topic in different lung cancer typing (NSCLC and SCLC), which was more specific to some extent. We concluded an amount of potential proteomic biomarkers and some related pathways, which might promote further clinical validation and provide insights and direction for future research.

## Author contributions

BL and ZZ had the idea for the article. ZX, YC and XZ performed the literature search and data analysis. JW drafted and critically revised the work. All authors contributed to the article and approved the submitted version.

## Conflict of interest

The authors declare that the research was conducted in the absence of any commercial or financial relationships that could be construed as a potential conflict of interest.

## Publisher’s note

All claims expressed in this article are solely those of the authors and do not necessarily represent those of their affiliated organizations, or those of the publisher, the editors and the reviewers. Any product that may be evaluated in this article, or claim that may be made by its manufacturer, is not guaranteed or endorsed by the publisher.

## Glossary

**Table:**

A1AT	alpha-1 antitrypsin
AMBP	alpha-1-microglobulin/bikunin precursor
APCS	serum amyloid p component
ARCN1	archain 1
AURKA	aurora kinase A
BCL6	B cell lymphoma 6
C4BPA	C4b-binding protein alpha chain
C9	complement component 9
Cdc42	cell division cycle 42
CDH1	E-cadherin
CDH3	cadherin-3
CEA	carcinoembryonic antigen
c-MET	mesenchymal-epithelial transition factor
CRP	C-reactive protein
CTSD	cathepsin D preproprotein
CYFRA	cytokeratin-19 fragment
DDR2	discoidin domain-containing receptor
DDX56	DEAD box polypeptide 56
EGFR	epidermal growth factor receptor
ERO1L	ERO1-like protein alpha
FGFR1	fibroblast growth factor receptor 1
GLB1	Galactosidase beta 1
GNA1	glucosamine 6-phosphate N-acetyltransferase 1
GPX4	glutathione peroxidase 4
GRP78	glucose-regulated protein 78 kDa
GSK3	glycogen synthase kinase 3
GSTM1	glutathione S-transferase M1
ETHE1	ETHE1 persulfide dioxygenase
HGF	hepatocyte growth factor
HRas	HRas proto-oncogene, GTPase
HSP	heat shock protein
KMT9	lysine methyl transferase 9
LRG1	leucine-rich alpha-2-glycoprotein
LRRC8A	leucine-rich repeatcontaining 8A
MACF-1	microtubule-actin crosslinking factor 1
MCL-1	myeloid cell leukemia-1
MMP-9	matrix metalloproteinase-9
MPS1	monopolar spindle 1
NARS	asparaginyl-tRNA synthetase, cytoplasmic
NR0B1	nuclear receptor subfamily 0 group B member 1
NRF2	nuclear factor erythroid 2 (NF-E2)-related factor 2
NSD	nuclear receptor-binding SET domain protein
p53	tumor protein p53
PABPC4	polyadenylatebinding-protein 4
PAI-1	plasminogen activator inhibitor-1
PARP	poly polymerase
PCBD1	pterin-4 alpha-carbinolamine dehydratase 1
PDIA	protein disulfide isomerase
PDPK1	3-phosphoinositide-dependent protein kinase 1
PGM1	phosphoglucomutase 1
PKA	protein kinase A
PLK1	Polo-like kinase 1
PON1	serum paraoxonase 1
PPA2	pyrophosphatase 2
PSMC5	proteasome 26S subunit ATPase 5
PYGB	glycogen phosphorylase B
QSOX1	quiescin sulfhydryl oxidase 1
Rac1	Ras-related C3 botulinum toxin substrate 1
RBP	retinol binding protein
RCC1	regulator of chromosome condensation
RhoA	Ras homolog family member A
RhoGDIα	Rho GDP dissociation inhibitor-α
RPS25	ribosomal protein S25
SCC	squamous cell carcinoma antigen
SCCA1	squamous cell carcinoma antigen 1
SCCA2	squamous cell carcinoma antigen 2
sCD40	soluble cluster of differentiation 40
SERPINA4	kallistatin
sFas	soluble Fas
SFK	SRC family kinases
SRI	sorcin
SUMO1	small ubiquitin-like modifier 1
TARS	threonine-tRNA ligase
TBK1	TANK-binding kinase 1
Tspan8	tetraspanin-8
TUBB3	βIII-tubulin
TyrRS	tyrosyl-tRNA synthetase
USP9X	ubiquitin-specific peptidase 9 X-linked
VTN	vitronectin
